# An Evaluation of Lymphedema Using Optical Coherence Tomography: A Rat Limb Model Approach

**DOI:** 10.3390/diagnostics13172822

**Published:** 2023-08-31

**Authors:** V. V. Nikolaev, I. A. Trimassov, D. S. Amirchanov, E. A. Shirshin, N. A. Krivova, S. A. Beliaeva, E. A. Sandykova, Yu. V. Kistenev

**Affiliations:** 1 Laboratory of Laser Molecular Imaging and Machine Learning, Tomsk State University, 36, Lenin Ave., Tomsk 634050, Russia; vik-nikol@bk.ru (V.V.N.); i.a.trimassov@mail.tsu.ru (I.A.T.); denis.amirhanov@yandex.ru (D.S.A.); nakri@res.tsu.ru (N.A.K.); sofia.al.beliaeva@gmail.com (S.A.B.); sandykova@mail.tsu.ru (E.A.S.); 2 Faculty of Physics, Lomonosov Moscow State University, Moscow 119991, Russia; eshirshin@gmail.com

**Keywords:** lymphedematous tissue, lymphedema diagnosis, optical coherence tomography, histology, curve proximity factor

## Abstract

Lymphedema is a pathology caused by poor lymphatic flow which may lead to complete disability. Currently, precise, non-invasive techniques for quantifying lymphedema are lacking. In this paper, the results of an in vivo assessment of lymphedema via a developed small-animal model using the hindlimbs of rats and an optical coherence tomography (OCT) technique are presented. This model of lymphedema was based on a surgical lymph node resection and subsequent two-step X-ray exposure. The development of lymphedema was verified via the histological examination of tissue biopsies. The properties of the lymphedematous skin were analyzed in vivo and compared with healthy skin via OCT. The main differences observed were (1) a thickening of the stratum corneum layer, (2) a thinning of the viable epidermis layer, and (3) higher signal attenuation in the dermis layer of the lymphedematous skin. Based on the distribution of the OCT signal’s intensity in the skin, a machine learning algorithm was developed which allowed for a classification of normal and lymphedematous tissue sites with an accuracy of 90%. The obtained results pave the way for in vivo control over the development of lymphedema.

## 1. Introduction

Lymphedema is caused by poor lymphatic flow that leads to chronic tissue transformation and edema [1,2]. This pathology is progressive and can cause complete disability if left untreated. According to the World Health Organization, lymphedema affects 300 million people, and 96% of them are of working age [3,4].

Lymphedema is associated with inflammation, the hypertrophy of adipose tissue, and the expansion of fibrosis with irreversible structural tissue damage [1,5]. Lymphedema significantly changes the deep layers of the skin and affects both the epidermis and papillary dermis [5]. In lymphedema, non-enzymatic mechanisms of the breakdown of the structure of collagen are implemented which are associated with the formation of additional intermolecular bonds under the action of mechanical pressure (edema) [6]. Destructive processes are accompanied by changes in the length, thickness, and spatial orientation of collagen fibers [7]. The development of secondary lymphedema is also accompanied by changes in the levels of metalloproteinases (enzymes capable of destroying all types of extracellular matrix proteins) [8], which can lead to increased collagen and elastin cleavage. Elastin fibers play a crucial role in directing the flow of fluid to the lymphatic vessels in the skin, and their failure to properly support the skin and lymphatics has been linked to progressive lymphedema [9,10,11]. Studies [9,10,11] have also shown that a genetic disorder affecting elastin metabolism can lead to the improper functioning of lymphatic vessels and the swelling of the lower extremities.

**Small-animal models of lymphedema:**

A small-animal model of lymphedema can aid in achieving a deeper understanding of the mechanisms of this disease and the invention of new diagnostic and therapeutic methods. One of the first small-animal models of lymphedema using the hind limbs of rats appeared in 1958 [12]. To provoke lymphedema, an injection of kaolin was used, which was administered once or twice (the interval between injections was 54 days). This model caused a slight increase in the volume of the rats’ limbs (from 1.5 to 15%) compared to healthy limbs. Later, this approach was criticized due to the lack of clinical evidence of lymphedema and the small statistical difference in the limb volumes [13].

Another approach was based on the surgical resection of lymph nodes on the rats’ limbs [13,14,15]. Kanter et al. [13] used X-ray radiation therapy at a dose of 45 gray (Gy) after the surgical removal of the lymph nodes near the hind limb. A combination of inguinal lymph node resection and radiation therapy was shown to successfully induce limb lymphedema in rats [16]. It was noted that in the absence of soft tissue excision, lymphedema does not develop [14,17]. Mendez et al. [18] excised axillary lymph nodes to develop lymphedema on the forelimbs of rats. After the removal of the lymph nodes and rehabilitation for 66 days, the authors used Oxazolone, causing contact hypersensitivity due to an immunological response to a foreign chemical. They also used bleomycin, which aggravated extracellular matrix fibrosis [19]. The drawback of this approach, which was based on the introduction of substances into the extracellular matrix, is that the formation of fibrosis was not necessarily connected with the development of lymphedema. To guarantee the development of lymphedema after the surgical removal of the lymph node, the healing tissue was scratched every three days to prevent epithelization [20]. But this procedure also causes acute inflammation, which can be a reason for an increase in limb volume.

A stable, small-animal model of lymphedema that was based on surgical protocol was created by the authors of [21]. The authors used indocyanine green staining and then made a vertically oriented incision in the mid-groin and dissected a strip of skin and subcutaneous tissue that was 8–10 mm wide circumferentially to the fascia, after which the deep lymphatics and popliteal and inguinal lymph nodes were dissected. This approach caused a statistically significant increase in limb volume for at least 45 days; the results were confirmed histologically. The drawback of this method is that it required the removal of a sufficiently large area of tissue around the limb, which is not a typical mechanism for the development of lymphedema in humans.

A model of lymphedema was also developed using an excision from the base of the mouse tail [22]. The effectiveness of this approach has been demonstrated; however, the experiments were performed just for two animals.

Approaches to the development of lymphedema and their benefits and drawbacks are summarized in Table 1.

All the approaches described above make it possible to create lymphedema on the limbs of small animals; however, each of them has drawbacks. The presented models of the development of lymphedema are related to the first two–three months after the onset of its formation; however, in some cases, a weak statistical analysis prevented a critical analysis of the reliability of the described results. Moreover, in most of the articles, there is no information about the stability of the obtained results. The desired properties of a small-animal model of lymphedema are as follows:

(1) The model should be analogous to clinical situations for humans. A typical situation occurs when a lymph node rejection is combined with X-ray exposure. For example, the surgical treatment for breast cancer includes the removal of the limb lymph nodes, after which radiation therapy is used [26]. In clinical practice, more than 90% of cases of secondary lymphedema occur after the surgical treatment of cancer, combined with subsequent X-ray radiation therapy or as a result of trauma [27].

(2) A long enough period of rehabilitation is necessary to exclude acute inflammation and associated edema.

**Methods for lymphedema diagnostics:**

The following methods for diagnosing lymphedema are used in clinical practice [28]. Direct and indirect methods of lymphography are based on the injection of contrast agents directly into the lymphatic vessel or tissues [29]. The disadvantages are the invasiveness of the methods and the use of radiocontrast agents [30]. The quantitative assessment of edema is based on the registration of an increase in limb volume via the displacement method [31], the truncated cone method [32], or optoelectronic pyrometry [33]. Until recently, the displacement method was the “gold standard” for diagnosing lymphedema [33]. The usefulness of this method for the early detection of lymphedema is questionable because the latent phase of lymphedema precedes edema [34]. Medical imaging tools like ultrasound imaging, magnetic resonance imaging, and computed tomography are used to diagnose lymphedema [35]. However, these methods are rather complicated or have low levels of specificity [34].

To study the structures and spatial distributions of collagen and elastin, imaging methods with a spatial resolution of units of microns are required. In practice, non-invasive methods are preferred. Among the variety of methods of optical microscopy, two-photon microscopy, optical coherent microscopy, and coherent anti-Stokes Raman spectroscopy are currently used for this task.

Fluorescent imaging is an alternative to direct and indirect lymphography. Instead of radiocontrast agents, fluorescent agents are injected into the lymph and then visualized under NIR excitation. Then, with the help of special cameras, the speed of the lymph’s movement and the penetration of fluorescent dyes into the interstitial space are evaluated. The disadvantage of this method is the necessity of the invasive introduction of agents into the lymphatic vessels [36].

Two-photon microscopy (TPM) is a non-invasive optical diagnostic method. This method allows for the instant in vivo visualization of tissues at the subcellular level. This method is based on the autofluorescence of endogenous fluorophores, for example, elastin, and second harmonic generation (SHG) from tissue components, for example, collagen [26]. This approach allows one to visualize the structural and functional features of tissue without the use of special labels. In [26], TPM and machine learning (ML) were used to diagnose lymphedema in humans. The measured SHG images made it possible to assess the structures of the collagen and elastin fibers in the affected tissues. The structurse of the collagen and elastin fibers in the diseased tissue were compared with the structure of healthy tissue. A gradient analysis using the Sobel operator, the Canny edge detector, a morphology-based edge detector, and a logarithmic operator were applied to quantify the collagen disorganization. As a result, a predictive model based on the group classification of the SHG image textures and the application of the voting scheme provided an accuracy of 96% in diagnosing lymphedema [26]. However, despite its effectiveness, TPM is a complicated and expensive method [26,37].

OCT is based on the phenomenon of light reflecting from the layers of material in a sample. OCT is a potentially effective method for the diagnosis of lymphedema [22]. OCT is a non-invasive optical imaging technique which allows one to obtain two-dimensional cross-sectional images of a sample in real time and three-dimensional images of a sample in volume with micron-level resolution. The OCT method has long been widely used in ophthalmology to study eye structures in vivo and to diagnose macular pathologies [38,39,40,41]. With the development of this method, applications emerged for the diagnosis of skin diseases, oncology, and vascular diseases [42,43,44]. Micrographic surgery for basal cell carcinoma using OCT was conducted [43] in which OCT was used to validate the clinically assessed boundaries of the micrographic surgery in order to reduce the excision area without affecting the areas of normal skin. The advantages of OCT are the ability to obtain the structural features of tissue at a depth of up to 2–3 mm, its non-invasiveness, and its scanning speed. In summary, the OCT method, in contrast to the TPM method, has a larger depth of penetration into the tissue and a lower price but a lower resolution. The usefulness of the OCT approach was shown in a recent study of rat tails used as a model of lymphedema; however, the use of this approach for the diagnosis of lymphedema remains insufficiently studied [22].

The purpose of this article is (1) to create a model of lymphedema which would be a reliable prototype of the emergence and development of human lymphedema implemented over a long period, combining the resection of lymph nodes with repeated radiation therapy and (2) its diagnosis using OCT.

## 2. Materials and Methods

### 2.1. Object of Study

The study was conducted on male Wistar rats aged 8–10 weeks (weight 200–250 g), using 16 animals in total. The rats were kept in an isolated and ventilated room in the vivarium of the Institute of Biology and Biophysics of Tomsk State University (Russia). The room temperature was 20 ± 2.0 °C, the air humidity was 60%, and the illumination followed a 12 h light/12 h dark cycle All animals were marked and kept for 7 days in quarantine, with 5 rats in one cage, with free access to water and food (a standard diet for rats). The study protocol was approved by the Bioethics Committee of the National Research Tomsk State University (extracted from Protocol No. 1 of the meeting of the Bioethics Committee of the National Research Tomsk State University, dated 23 November 2018).

### 2.2. Protocol for the Development of Lymphedema in Rats 

**Figure 1:**
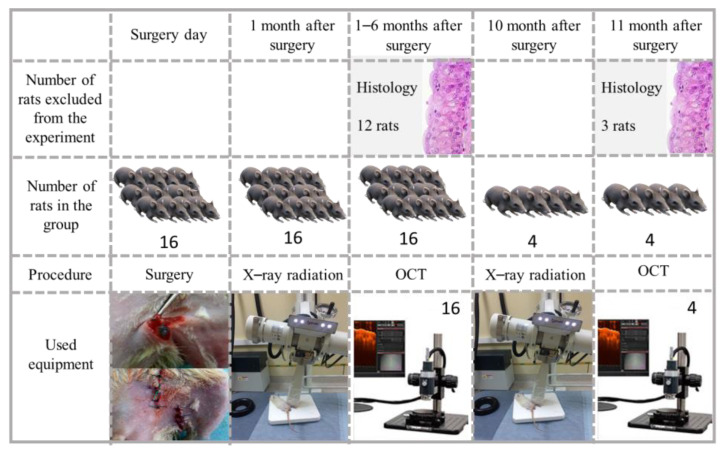
Schematic presentation of the developed experimental protocol.

Our protocol for the development of lymphedema combines a lymph node surgical resection with X-ray radiation therapy repeated over a long period of time (Figure 1). The popliteal ganglia (Inn. popliteus) were removed on one limb under anesthesia (Zolazepam + Xylazine). The dosage was calculated using the weight of the animal: 2 mg/kg of Zolazepam and 1.5–2 mg/kg of Xylazine (Zolazepam + Xylazine 1:1 (0.02 + 0.02 mL)). The other limb was used as a control (healthy tissue). One month after the operation, X-ray radiation was applied to the region of the hind limb. This interval was determined by the fact that postoperative tissue edema is usually observed within 3 weeks from the surgical event.

The X-ray radiation exposure was implemented using an X-Strahl 200 apparatus (Xstrahl Ltd., Walsall, UK). The exposure dose was 20 Gy over 20 min, chosen in accordance with the modified and supplemented Nominal Standard Dose (NSD) system of time–dose fractionation for short-range X-ray therapy. The X-ray radiation exposure was repeated 10 months after the operation.

The hind limb circumference was measured at five points. The first measurement point was marked 1 cm above the calcaneus, and the remaining points were placed further with steps of 0.5 cm. As a result, the median, upper, and lower quartiles were calculated for each measurement. The volume was calculated using the truncated cone formula. The calculation was carried out as follows: the volume of the lower leg was calculated from the first to the third point and from the third to the fifth point, and then the resulting volumes were added. Significant changes in volume were not observed [25]. This fact is probably associated with the presence of acute edema within a month after the operation and its subsequent compensation. The latter means that chronic tissue transformation could occur without evident changes in the volume of the rat limbs.

### 2.3. Histology

For histological analysis, skin biopsies (of the dermis and hypodermis) of the inner surface of the posterior limb were taken. The following standard protocol for histological examination was used: fixation in a 10–12% neutral formalin solution (BioVitrum, Saint-Petersburg, Russia) for 24 h, followed by washing, dehydration, and paraffinization. Paraffin sections with a thickness of 5–7 μm (ACCU-CUT SRM ™ 200 “Sakura” Rotation microtome of Tokyo, Japan,) were stained with hematoxylin and eosin [45]. Photos were taken at a magnification of 100 and 200 for 5–10 random fields of vision using a Canon Powershot G10 digital camera (Canon, Tokyo, Japan).

### 2.4. Optical Coherence Tomography

This study was carried out using a GANYMEDE-II OCT device (ThorLabs, Newton, MA, USA). The device was equipped with a super-luminescent diode (the central wavelength was 930 nm). The axial scanning speed was 1.5–80 kHz, and the maximum sensing depth was 2.9 mm with an axial resolution of up to 5.5 microns (in air). The OCT measures the profile of the intensity of backscattered light over the depth of the tissue. The profile at one point is called the A-scan, the profile across the surface is the B-scan, and the profile in the volume is the C-scan.

**Figure 2:**
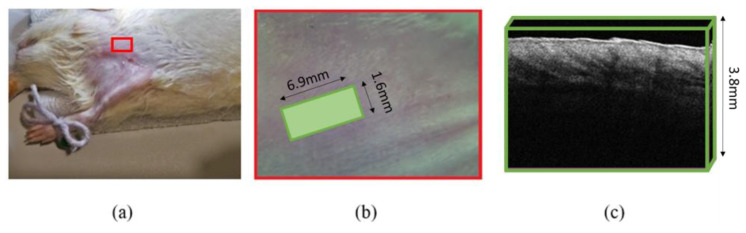
Photo of rat fixation during the OCT study with highlighted area of interest (**a**), a scanning area (**b**), and an example of a C-scan image (**c**).

Figure 2 shows some details of the implementation of the OCT study in the rat tissues. The C-scans were measured in a 3D matrix at sizes of 300 × 695 × 1253 pixels.

Three C-scans were measured for each rat limb just after the surgery. Also, 36 and 9 C-scans were measured for the same limbs after the first and the second X-ray radiation exposures, respectively. The same number of images was measured for healthy limbs. Differing amounts of data were due to the withdrawal of some animals from the experiment (Figure 1).

**Figure 3:**
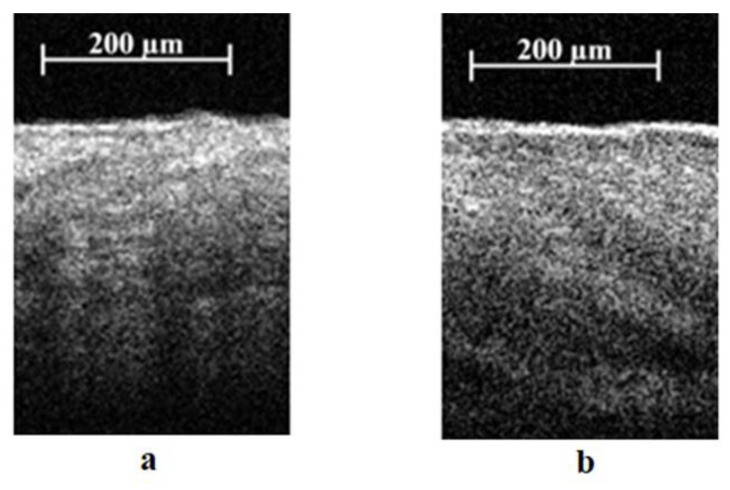
An example of B-scans of healthy tissue (**a**) and lymphedematous tissue (**b**) from the developed model.

All C-scans were divided into 300 B-scans. Each B-scan was viewed, and areas with high levels of slope or hair were excluded from consideration. A-scans were obtained as averaged B-scans after removing low-quality or defective areas. A total of 1962 A-scans were analyzed for the healthy group, 1623 for the lymphedematous rats after the first X-ray radiation exposure, and 532 after the second X-ray radiation exposure. An example of images of healthy and lymphedematous skin is shown in Figure 3. A Gaussian filter was applied to the A-scans (with a window width of 9 and a sigma of 1.5).

**Figure 4:**
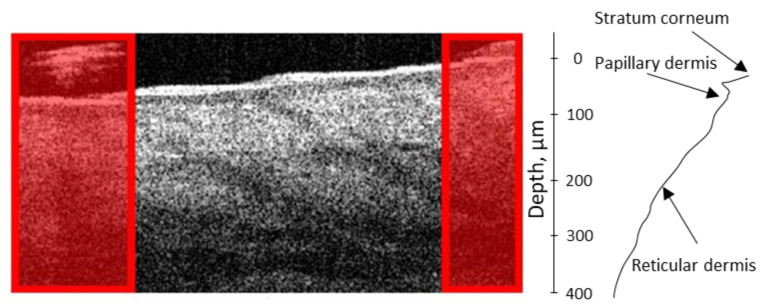
An example of filtering out inappropriate areas of the OCT image. Examples of areas excluded from the analysis are marked in red.

In the first stage of data preprocessing, the removal of outlier data was carried out manually (see Figure 4).

Then, a Gaussian filter with a window size equal 9 and a sigma of 1.5 was applied over the 3 × 3-pixel region to reduce noise. In this case, a Gaussian filter was used to clean the data before fitting. The filter parameters were chosen to reduce the spread in the data after the approximation. After that, the dependence of the intensity of the OCT signals on depth was evaluated.

### 2.5. Machine Learning

**Figure 5:**
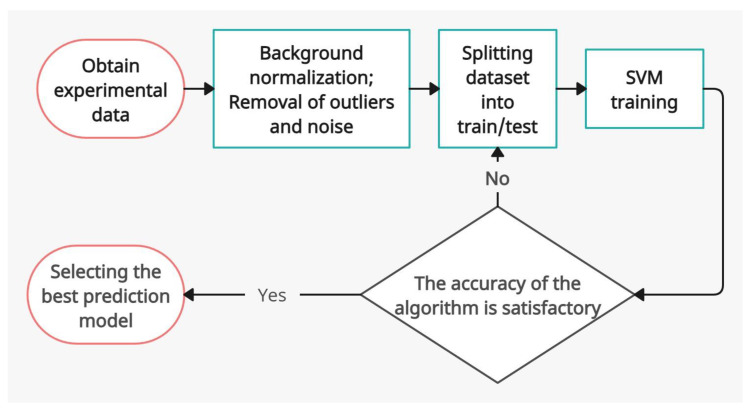
The ML pipeline.

A machine learning (ML) algorithm for the diagnosis of lymphedema using OCT data was implemented. The task of a two-class classification was set in which one class corresponded to healthy tissue and the other corresponded to lymphedematous tissue. A Support Vector Machine (SVM) with a Radial Basis Function (RBF) kernel was used for the binary classification. The ML pipeline scheme is shown in Figure 5.

The OCT data preprocessing included (i) “air removal” so that the first element in the array corresponded to the position of the stratum corneum (in other words, the black area at the top in Figure 3 was removed) and (ii) median filtration (using a window size equal to 3). Next, the data were divided into groups. Three training data sets were created containing 80% of the original data. The rest of the data were used for model testing. Data collected from one individual were not included in the training and test sets at the same time.

## 3. Results

**Figure 6:**
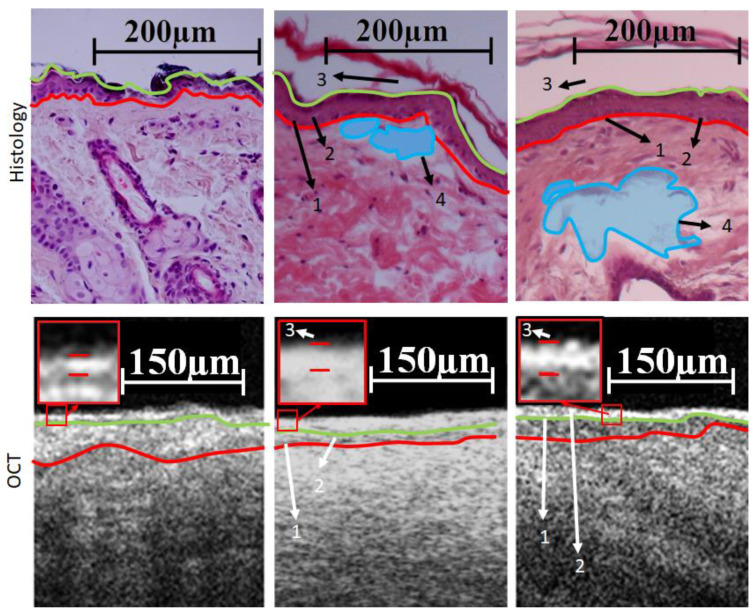
Comparison of histological and OCT images for the control limb and the lymphedematous limb after the first and second exposures to X-ray radiation. Here, “1” shows the smoothing of the papillary layer of the dermis, “2” shows the thinning of the viable epidermis, “3” shows the thickening of the stratum corneum, and “4” shows the presence of fluid in the tissue. The red and green curves correspond to the dermal–epidermal junction and lower border of the stratum corneum, respectively.

The examples of the recorded histopathologic and OCT images for the control limb and the lymphedematous limb after the first and second exposures to X-ray the second X-ray radiation are shown in Figure 6.

The following morphological changes can be observed upon comparing the histology and OCT images. First, a thinning of the epithelial layer (viable epidermis, “2”) and a thickening of the stratum corneum were observed after the first and the second exposures to X-ray radiation. Next, a smoothening of the dermal–epidermal junction can be seen for the irradiated skin (“4”), as well as the emergence of areas with increased water content (areas colored in blue).

Intravital measurements of the limb volume were also obtained. Skin volume measurements were carried out two weeks after the first and second X-ray radiation exposures for the control limb and the limb with lymphedema [25]. The difference in the volume of the limb after the first X-ray radiation exposure did not exceed 10%. This difference disappeared after 11 months. This fact is probably due to the presence of acute edema during the first month after surgery and its compensation, as well as a small fraction of muscles and tissues in the rat limb which were removed during surgery. Hence, it is likely that chronic tissue transformation does not necessarily have to be accompanied by a change in the volume of the rat limb.

**Figure 7:**
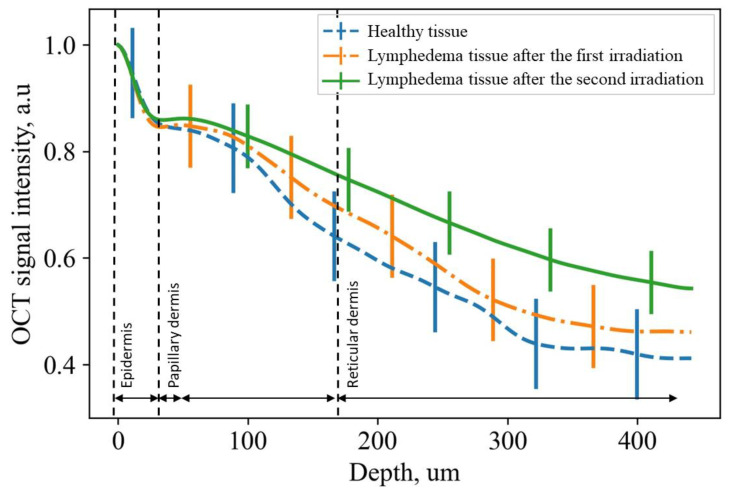
A-scans for healthy and lymphedematous tissues after the first and second X-ray radiation exposures.

According to the procedure described above, the dependence of the intensity of OCT signals on depth (average A-scan) was estimated (see Figure 7).

**Figure 8:**
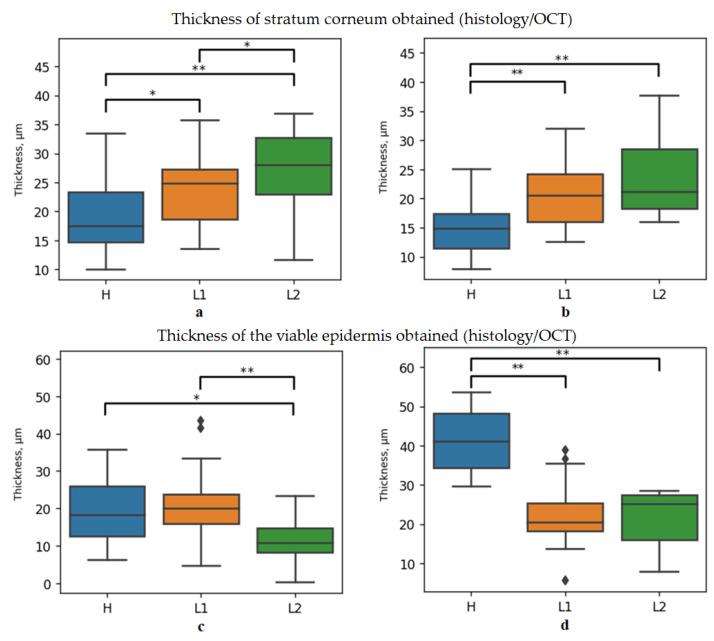
Boxplots of the thickness of the stratum corneum (histology (**a**), OCT (**b**)) and viable epidermis (histology (**c**), OCT (**d**)) skin layers for the healthy (H) and lymphedematous skin after the first (L1) and the second (L2) X-ray irradiation. * and ** correspond to *p*-value < 0.05 and *p*-value < 0.01, respectively.

On the averaged A-scan, the position of the local intensity minimum corresponds to the end of the stratum corneum and the beginning of the viable epidermis, and the position of the second maximum corresponds to the beginning of the dermis; therefore, the signal after the second peak was taken as a signal from the dermis. For the stratum corneum measured via histology and OCT, there are statistically significant differences the normal and lymphedematous tissues (see Figure 8a,b). It can be noted that while the histology data did not reveal significant changes in epidermal thickness following the initial radiation exposure, OCT was able to detect differences (see Figure 8c,d).

**Figure 9:**
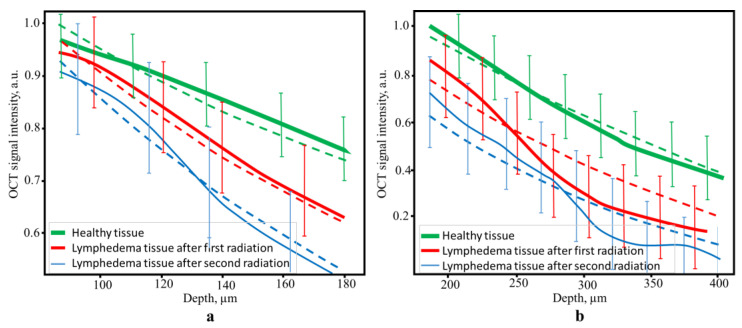
The experimental A-scans and their approximation via the formula $I=I_{0}\exp\left(-\mu z\right)+B$ for healthy and lymphedematous tissues after the first and second radiation exposures for the papillary dermis (80–180 µm) (**a**) and reticular dermis (180–400) µm (**b**) depths (the dotted line is the corresponding experimental curve approximation).

We further analyzed the difference in light attenuation between the normal and irradiated skin by evaluating the extinction coefficient (which is in fact a sum of the reflection, absorption, and scattering coefficients). The extinction coefficients of the dermis were estimated from the exponential law at a depth interval of 0.12–0.4 mm using the formula $I=I_{0}\exp\left(-\mu z\right)$, where $I_{0}$ is the intensity of light at the entrance to the substance, μ is the extinction index, and z is the depth to which the light propagates (Figure 9).

For the approximation, the averaged values of the OCT signal intensity were processed by the noise filter and normalized to the maximum. Averaging was carried out over 100 points. The obtained mean values of the extinction coefficient and its variance are presented in Table 2.

The increase in the dermis extinction coefficient is among the first indicators of the development of lymphedema. As can be seen from Table 2, changes in the optical properties of the dermis beginning from a depth of 100 µm are significantly different for the normal and irradiated skin. These changes are probably associated with the formation of fibrous tissue in the limbs. At a depth of more than 380 µm, the OCT signal is essentially decreased, and any differences between lymphedematous and healthy tissues cannot be established.

The curve proximity factor (CPF) *S* [46] was used to compare quantitative differences in the A-scans for healthy and lymphedematous tissues:

(1) $$S=1-\frac{\sum_{i}\left|X_{i}-Y_{i}\right|}{\frac{1}{2}\sum_{i}\left|X_{i}+Y_{i}\right|},$$

where *X_i_* and *Y_i_* represent the intensity of the OCT signal for a definite depth from the lymphedematous and the healthy skin, respectively. The closer the CPF value is to 1, the higher the similarity of the skin is to lymphedematous and healthy skin. The CPF values are shown in Table 2.

As can be seen, a significant difference in the distribution of the extinction coefficient with depth was obtained between the healthy tissue and the lymphedematous tissue after the second X-ray irradiation.

The potential of ML for distinguishing between different skin groups was also estimated. Accuracy was defined as the proportion of correct predictions made by the model as follows:

(2) $$Accuracy=\frac{Number\;of\;correct\;predictions}{Total\;number\;of\;predictions}\;.$$

During the training process, the parameters for pre-processing were adopted. The SVM algorithm was employed, and the findings are presented in Table 3. Here, 80% of examples were used for training, and the rest were used for testing the model.

Notably, the accuracy of the algorithm was higher for the data obtained after the second exposure to X-ray radiation. This could be caused by the fact that the effects of the disease were more pronounced at this stage. The results of the SVM classification are quite promising for the diagnosis of lymphedema using OCT data.

## 4. Discussion

In this study, a model of lymphedema combining lymph node resection with repeated X-ray radiation therapy was suggested. The main difference of this model from the previously proposed ones (Table 1) is its relevance to the onset of clinical human lymphedema following tissue cancer therapy as it combines a lymph node resection with repeated X-ray radiation therapy and is implemented over a long period.

The developed model of lymphedema was based on the hypothesis that the pathogenesis of this disease requires a trigger—the impact of an external factor (provocation). For example, repeated irradiation can serve as such a factor. To exclude other factors (e.g., inflammation and skin trauma), the first X-ray radiation exposure was carried out 1.5 months after the surgical operation, and the second X-ray radiation exposure was carried out 10 months after the surgery. All measurements were performed two weeks after X-ray radiation exposure (see Figure 1).

A histological examination showed changes in the skin that are characteristic of lymphedema. A smoothing of the contour of the papillary layer for the lymphedematous tissue was observed, as was hyperkeratosis, and in the reticular layer of the dermis, areas of the accumulation of tissue fluid were noted. Lymphatic hemangiopathy was also detected, which is characterized by an increase in the density of the vascular component, represented by capillaries. These changes were observed in all rats in the groups after exposure to X-ray radiation.

CPF and the extinction coefficient showed significant differences between the lymphedematous tissues after the second exposure to X-ray radiation and the healthy tissue, while the differences after the first exposure to X-ray radiation were not so pronounced. This fact indirectly indicates a change in the structure of the dermis, i.e., the disorganization of collagen fibers, as well as the destruction of elastin [26]. In accordance with this, the ML algorithm for the classification of the state of lymphedema based of an analysis of the OCT intensity profiles yielded an accuracy of ~90%, also illustrating the significant differences in light attenuation for normal and affected skin areas.

## 5. Conclusions

In this study, a model of lymphedema and a method for diagnosing lymphedema using in vivo OCT were suggested.

The model of lymphedema was based on combining lymph node resection with repeated X-ray radiation therapy, creating a stable lower limb in rats. This approach has been partly applied in the literature; however, only a single exposure to X-ray irradiation and a short rehabilitation time were used. According to the histology data obtained, a tissue transformation typical for lymphedema was observed; however, no large edema characteristic of lymphedema could be seen.

The CPF coefficient was applied to the OCT data, which allowed for a quantitative comparison of the OCT signal curves in the tissue. The extinction coefficient also indicated changes in the organization of the dermis during the development of lymphedema.

Thus, this work shows the possibility of the in vivo study of the state of the papillary dermis in lymphedema at the microscopic level. The obtained results enable a non-invasive diagnosis of the disease in its early stages, which is important for early and effective therapy. Mathematical approaches to the analysis of OCT images that are based on quantitative parameters allow for express diagnostics, which can later be introduced into clinical practice.

## Figures and Tables

**Table 1:** Models of lymphedema induction and methods of provoking the disease.

Method			Temporal Interval for the Development of Lymphedema	Benefits	Drawbacks	Refs.
RT	Surgery	A Chemical Injection				
-	-	Kaolin was injected to create LE	From 14 to 133 days	Surgery or X-ray radiation exposure is not necessary.	An absence of clinical signs; small statistical difference in the volume of the limbs.	[12]
RT at a dose of 45 Gy	Surgical removal of LNs	-	Several days	Simple and clear technique with low mortality (<8%).	Soft tissue excision required.	[13]
RT with a single dose of 15 Gy	Excision of the superficial inguinal and popliteal LNs, and adjacent LV	-	30 days	MRI verification.	No differences upon macroscopic inspection of the legs. No histological verification.	[14]
-	Excision of the skin and subcutaneous tissue 8–10 mm wide, followed by excision of the deep LV	-	30 days	Clinically relevant second stage of LE.	The removal of a large volume of soft tissue.	[21]
RT at a dose of 22.7 Gy	Removal of popliteal and inguinal LNs; excision of inguinal adipose tissue	-	56 days	Violation of lymph outflow verified via lymph fluoroscopy; an increase in limb volume.	Excised inguinal adipose tissue required.	[23]
RT at a single dose of 20 Gy	Circumferential skin excision on the 10th day after RT	-	20 days	An increase in limb thickness of about 20%; lymphedema verified by histology.	A circumferential skin excision was performed at the level of the inguinal crease.	[24]
RT at dose of 20 Gy	Resection of the popliteal LN, followed by lymph dissection	-	30 days	Small tissue damage, close to the clinical manifestation of LE in humans.	The volume of the limb does not increase.	[25]

Abbreviations: LE—lymphedema; LN—lymph node; RT—radiotherapy; LV—lymph vessel.

**Table 2:** Extinction coefficient and CPF values for healthy and lymphedematous tissues.

Tissues	Tissue Extinction Coefficient		CPF
	Depths: 80–180 µm	Depths: 180–400 µm	
Healthy tissue	78.912 ± 15.343	100.813 ± 17.000	
Lymphedematous tissue after the first X-ray therapy	57.282 ± 14.050 *	74.473 ± 13.277 *	0.05 ± 0.04
Lymphedematous tissue after the second X-ray therapy	52.221 ± 16.050 *	71.539 ± 14.872 *	0.31 ± 0.18 *

Comment: *—indicates the statistical significance of the difference between the analyzed value and the same value for a healthy tissue (Mann–Whitney test, *p* < 0.05).

**Table 3:** Results of the classification of healthy and lymphedematous tissue samples in the testing set of the OCT intensity profiles.

	Algorithm Accuracy, %	
	Mean	STD
Lymphedematous tissue after the first X-ray therapy	89.1	11.5
Lymphedematous tissue after the second X-ray therapy	90.6	9.4

## Data Availability

The data presented in this study are available upon request from the corresponding author. The data are not publicly available due to privacy or ethical restrictions.
